# Dietary Supplementation of Protease and/or Amylase Improves Growth Performance, Digestive Function, and Intestinal Health of Weaned Piglets

**DOI:** 10.3390/ani16162538

**Published:** 2026-08-14

**Authors:** Runzi Tang, Jiaqi Chen, Binke Chen, Jiayue Zhao, Wenzi Wu, Hanyue Zhang, Aikun Fu, Xiuan Zhan

**Affiliations:** Key Laboratory of Animal Nutrition and Feed Science in East China, Ministry of Agriculture and Key Laboratory of Molecular Animal Nutrition (Zhejiang University) Ministry of Education, Institute of Feed Science, College of Animal Sciences, Zhejiang University, Hangzhou 310058, China; 22317093@zju.edu.cn (R.T.); 12417005@zju.edu.cn (J.C.); 12617017@zju.edu.cn (B.C.); 22317082@zju.edu.cn (J.Z.); 12517006@zju.edu.cn (W.W.); 22517016@zju.edu.cn (H.Z.); aikunfu@zju.edu.cn (A.F.)

**Keywords:** protease, amylase, weaned piglets, growth performance, intestinal health

## Abstract

A total of 96 26-day-old weaned piglets were randomly assigned to four groups with three replicates per group: (1) CON group (basal diet), (2) PRS group (protease supplementation), (3) AMS group (amylase supplementation), and (4) combined protease and amylase group (PAS group) (protease and amylase supplementation). Compared to the CON group, AMS group reduced feed-to-gain ratio (*p* < 0.05). Additionally, all treatments increased ether extract digestibility (*p* < 0.05). The PRS and AMS groups reduced serum urea nitrogen level (*p* < 0.01). PAS group increased total protein, and decreased total cholesterol (*p* < 0.05). PRS and/or AMS supplementation increased serum glutathione peroxidase. IgM and IgG were higher in the AMS group. Furthermore, levels of D-Lactate and diamine oxidase were reduced (*p* < 0.05). PRS group exhibited increased fecal acetic acid and valeric acid levels (*p* < 0.05). Microbiota analysis showed that PRS or/and AMS supplementation enriched bacteria, including Anaerovoracaceae and *Blautia.* Metabolomics analysis revealed that health-promoting differential metabolites, including S-nitrosoglutathione and kynurenic acid, were further enriched, indicating enhanced immune regulation and intestinal homeostasis. Taken together, PRS or/and AMS supplementation can effectively promote nutrient digestion, intestinal microbiota structure and metabolism, and enhance intestinal barrier, thereby improving growth performance in weaned piglets.

## Simple Summary

Early weaning often leads to underdeveloped digestive systems, intestinal barrier damage, and microbial dysbiosis in weaned piglets, consequently restricting growth performance. Protease (PRS) and amylase (AMS) can effectively compensate for the deficiency of endogenous digestive enzymes and improve growth performance, intestinal health, and microbiota in weaned piglets. By supplementing a basal diet with PRS or/and AMS, we successfully reduced the feed-to-gain ratio, improved nutrients digestibility, enhanced antioxidant and immune capacity, and protected intestinal barrier integrity of piglets. Furthermore, it optimized intestinal microflora structure and metabolome, which can be used as exogenous feed enzyme preparations for weaned pig production.

## 1. Introduction

Although early weaning is widely adopted in intensive pig production to enhance sow reproductive efficiency, it imposes one of the most severe physiological challenges during the pig life cycle [1]. During this transitional period, piglets simultaneously experience separation from the sow, mixing with unfamiliar pen mates, environmental changes, deprivation of milk-derived nutrients and immunological factors, and an abrupt shift to solid plant-based feed [2,3,4]. Consequently, these factors lead to reduced feed intake, impaired nutrient absorption, and increased susceptibility to intestinal inflammation and pathogen colonization, diarrhea and transient growth depression [5,6,7]. Previous studies have shown that weaning stress can lead to compromised intestinal morphology, diminished digestive capacity, and microbial dysbiosis [8,9,10]. Therefore, improving digestive adaptation and intestinal resilience in weaned piglets remains a central objective in high-efficiency swine production systems.

Exogenous feed enzymes are widely used in livestock production to degrade specific feed substrates. Although various enzymes have been extensively studied in pigs and poultry, their efficacy is strongly influenced by host physiology, dietary matrices, and gastrointestinal physicochemical conditions [11,12,13,14,15]. In practical diets for weaned piglets, PRS and AMS are particularly relevant, as crude protein (CP) and starch constitute the primary nutrient fractions, and weaned piglets are prone to insufficient CP and carbohydrate digestion. Stable PRS can degrade resistant proteins to enhance CP digestibility, reduce antigenicity, and minimize nitrogen excretion, whereas stable AMS can accelerate starch hydrolysis to maximize digestible energy and modulate carbohydrate availability [16,17,18,19,20,21].

Several studies have demonstrated that PRS supplementation enhances intestinal health, nutrient digestibility, and growth performance in pigs. However, the magnitude and consistency of these effects are influenced by factors including enzyme source, dietary inclusion level, diet composition, environment and the duration of supplementation [22,23,24,25,26,27,28,29,30]. Similarly, the efficiency of AMS supplementation relies on feed particle size and cereal-based diet conditions [16,17,18,19,20,21]. However, the specific co-supplementation of thermostable protease and amylase in weaned piglet diets remains largely unexplored.

Despite the limited number of studies evaluating the combined supplementation of PRS and AMS in weaned piglet diets, the present study investigated the individual and combined effects of these enzymes on growth performance, nutrient digestibility, intestinal barrier function, and gut microbiota composition. By elucidating the physiological and microbial responses to PRS and AMS supplementation, this study provides experimental evidence to support their effective dietary application for alleviating weaning stress and improving the health and growth performance of weaned piglets.

## 2. Materials and Methods

### 2.1. Reagents

A commercial mono-component protease (ProAct™, DSM, Shanghai, China) with an activity of 75,000 PROT/g and a commercial amylase (HiStarch™, DSM, Shanghai, China) with an activity of 600 KNU/g were used in this study. According to the manufacturer’s specifications, the protease retained 94% of its initial activity after exposure to 90 °C for 90 s and 93% after incubation at pH 3.0 in the presence of pepsin for 45 min. Under the same conditions, the amylase retained 91% and 82% of its initial activity, respectively. These data indicate that both enzymes exhibit substantial stability under feed-processing and simulated gastric conditions. Thermostability data of the enzymes were provided by the manufacturer.

### 2.2. Animals and Treatment

A total of 96 healthy Duroc × Landrace × Yorkshire weaned piglets at 26 d of age with similar initial body weight (10.46 ± 0.16 kg) were selected and randomly assigned into four groups: (1) CON group, basal diet; (2) PRS group, basal diet supplemented with 150 mg/kg protease; (3) AMS group, basal diet supplemented with 133 mg/kg amylase; (4) and PAS group, basal diet supplemented with 150 mg/kg protease and 133 mg/kg amylase. Every treatment group consisted of three replicates, with eight piglets each, and piglets were allocated with a balanced sex distribution (but limited by the relatively small number of replicates). Piglets were managed under routine feeding conditions and provided with feed and water ad libitum throughout the 42-day experiment. Piglets were housed in fully slatted concrete pens. Each pen measured 3.6 m × 2.5 m, with a stocking density of 1.125 m^2^ per pig. Average room temperature was maintained at 24.5 ± 2 °C throughout the experimental period. All piglets received standard commercial vaccination programs against swine fever and foot-and-mouth disease. No therapeutic antibiotics or pharmacological growth promoters were administered to animals during the trial. The basal diet (Table 1) was formulated based on the nutritional requirements for piglets according to NRC 2012 guidelines.

### 2.3. Digestibility Trial and Sample Collection

During day 36–39 of the feeding trial, apparent nutrient digestibility was determined using acid-insoluble ash as an internal marker. Feed and fecal samples were collected daily over four consecutive days from each replicate, with four subsamples obtained per replicate per day. All subsamples were stored individually without pooling until analysis. For the determination of nutrient digestibility, fresh fecal samples were acidified with 10% hydrochloric acid (10 mL per 100 g feces) to fix nitrogen and were preserved at −20 °C. The remaining non-acidified feces were further divided into two aliquots: one aliquot was stored at −20 °C for analyses of volatile fatty acids, and the other was stored at −80 °C for gut microbiota and metabolomic analyses to prevent microbial and metabolite degradation.

On the last day of the experiment, piglets were fasted and weighed. Four piglets from each replicate and balanced sex distributions were randomly selected for blood collection from the jugular vein. Blood samples were incubated at 37 °C for 10 min, centrifuged at 1800× *g* for 20 min, and serum was collected and stored at −20 °C until analysis. Fresh rectal feces were collected aseptically, immediately stored in liquid nitrogen and then transferred to −80 °C.

### 2.4. Growth Performance and Apparent Digestibility

Body weight (BW) was recorded on days 1 and 42 to determine cumulative body weight gain and overall average daily gain (ADG) during the 42-day experimental period. In order to minimize the stress experienced by the animals after weaning, no mid-term body weight measurements were included in the research design. For digestibility analysis, fecal samples were dried at 65 °C to constant weight, equilibrated for 24 h, ground and passed through a 40-mesh sieve.

(1) $$(g/d)\mathrm{ADG}=\frac{\mathrm{Final}\;\mathrm{average}\;\mathrm{body}\;\mathrm{weight}\;-\;\mathrm{Initial}\;\mathrm{average}\;\mathrm{body}\;\mathrm{weight}\;}{N\mathrm{umber}\;\mathrm{of}\;\mathrm{experimental}\;\mathrm{days}\;}$$

(2) $$F/G=\frac{\mathrm{Total}\;\mathrm{feed}\;\mathrm{consumption}\;\mathrm{per}\;\mathrm{pen}\;}{\mathrm{Total}\;\mathrm{weight}\;\mathrm{gain}\;\mathrm{per}\;\mathrm{pen}}$$

(3) $$(g/d)ADFI=\frac{\mathrm{Total}\;\mathrm{feed}\;\mathrm{consumption}\;\mathrm{per}\;\mathrm{pen}\;}{\mathrm{Number}\;\mathrm{of}\;\mathrm{surviving}\;\mathrm{pigs}\;\mathrm{in}\;\mathrm{the}\;\mathrm{pen}\;\times \;\mathrm{Number}\;\mathrm{of}\;\mathrm{experimental}\;\mathrm{days}\;}$$

Crude protein (CP), ether extract (EE), and crude fiber (CF) in feed and fecal samples were analyzed according to GB/T 6432-2018 [31] (Kjeldahl method), GB/T 6433-2025 [32], and GB/T 6434-2006 [33] (filtration method), respectively.

(4) $$\%\mathrm{Metabolism}\;\mathrm{rate}=100\;-(100\;\times \;\frac{\%\mathrm{acid}-\mathrm{insoluble}\;\mathrm{ash}\;\mathrm{in}\;\mathrm{feed}}{\%\mathrm{acid}-\mathrm{insoluble}\;\mathrm{ash}\;\mathrm{in}\;\mathrm{feces}}\;\times \;\frac{\%\mathrm{nutrient}\;\mathrm{in}\;\mathrm{feces}}{\%\mathrm{nutrient}\;\mathrm{in}\;\mathrm{feed}})$$

### 2.5. Determination of Serum Biochemical Parameters and Volatile Fatty Acids (VFAs)

Serum biochemical indices, including total protein (TP), total cholesterol (T-Cho), triglyceride (TG), high-density lipoprotein cholesterol (HDL-C), low-density lipoprotein cholesterol (LDL-C) and urea nitrogen (UN), were quantified using assay kits (Jiancheng, Nanjing, China) according to manufacturers instructions.

Serum antioxidant indices, including total antioxidant capacity (T-AOC), glutathione peroxidase (GSH-Px) and superoxide dismutase (SOD), and immunoglobulins including IgA, IgG and IgM were also determined using quantified assay kits (Jiancheng, Nanjing, China) according to manufacturer’s instructions.

Serum D-lactate, diamine oxidase (DAO) and endotoxin (ET) were measured as intestinal permeability markers, using assay kits (Jiancheng, Nanjing, China) according to manufacturer’s instructions.

VFAs were analyzed with gas chromatography. Briefly, 1 g of feces was dissolved in deionized water and mixed thoroughly. The mixture was centrifuged at 10,000× *g* under 4 °C for 10 min to obtain the supernatant. Then, supernatant was vortexed and allowed to rest for 30 min to acquire another supernatant. Next, 1 mL of the new supernatant was mixed with 0.2 mL of 25% phosphoric acid. The mixed liquid was stored at 4 °C for 30 min before being centrifuged. The finally collected supernatant was determined using a gas chromatograph. VFAs were quantified using a gas chromatograph (GC-2030, Shimadzu, Kyoto, Japan) equipped with a DB-FFAP capillary column (30 m × 0.25 mm × 0.25 μm). Nitrogen was used as the carrier gas. The oven temperature program was set as follows: initial temperature 40 °C held for 1 min, increased to 240 °C at a rate of 12 °C/min, and maintained at 240 °C for 4 min. The temperatures of injector and flame ionization detector (FID) were set at 230 °C and 250 °C, respectively. The injection volume was 1 μL with a split ratio of 10. External standards including acetic acid, propionic acid, isobutyric acid, butyric acid, isovaleric acid and valeric acid were applied for qualitative and quantitative analysis.

### 2.6. 16s rRNA-Based Microbiota Analysis

16S rRNA amplicon sequencing and analysis were performed by Majorbio Bio-Pharm Technology Co., Ltd. (Shanghai, China). Fecal microbial DNA was extracted and the V3–V4 region of bacterial 16S rRNA genes was sequenced on an Illumina MiSeq platform. Sequences were processed into amplicon sequence variants, annotated against the SILVA database, and analyzed for α-diversity, β-diversity and differentially enriched taxa.

### 2.7. Metabolite Extraction and Analysis

Fecal metabolomic analysis was conducted by Majorbio Bio Pharm Technology Co., Ltd. Each metabolite underwent normalization, and log-transformed data were used for statistical analysis. Four individual pigs were selected for 16S sequencing from the treatment group. Metabolic features were estimated according to the Human Metabolome Database. The R package ‘ropls’ (Version 1.6.2) was used to conduct principal component analysis (PCA) and orthogonal least partial squares discriminant analysis (OPLS-DA). Differential metabolites were screened using VIP > 1.5 and *p* < 0.05, annotated in public metabolite databases and enriched against KEGG pathways.

Spearman correlation analysis was performed to explore associations between differential bacterial genera and fecal metabolites.

### 2.8. Statistical Analysis

Data from the present study were subjected to two-way ANOVA using the general linear model in SPSS 25.0 (SPSS Inc., Chicago, IL, USA), the model included the main effects of dietary PRS level, dietary AMS level, and their interaction. Tukey’s post hoc test was used for pairwise comparisons among means. Results are presented as means ± standard deviation (SD), calculated from the means of experimental replicates. Differences were considered statistically significant at *p* < 0.05. Figures were generated using GraphPad Prism 10.1.2.

## 3. Results

### 3.1. Growth Performance

The effects of PRS or/and AMS supplementation on the growth performance of weaned piglets are presented in Table 2. A main effect of AMS was observed for F/G (*p* = 0.027), while no main effects of PRS or PRS × AMS interaction were detected for all measured growth indices. Initial BW, final BW, ADG and ADFI remained unaffected by treatments. The AMS group decreased the F/G compared with the CON group.

### 3.2. Apparent Nutrient Digestibility

As shown in Table 3, main effects of PRS (*p* = 0.041), AMS (*p* = 0.022), and PRS × AMS interaction (*p* = 0.035) were observed. Multiple comparison results indicated that diets supplemented with PRS or/and AMS improved the apparent digestibility of EE compared with the CON group. No main effects or interaction were detected for digestibility of CP and CF, while PRS showed a marginal trend for improving CP digestibility (*p* = 0.067).

### 3.3. Serum Biochemical Indices

Serum biochemical results are shown in Table 4. The main effects of PRS and AMS were detected for serum UN (*p* < 0.001), without interaction. Compared with the CON group, serum UN levels were decreased in all experimental groups. The PRS × AMS interaction was observed for TP (*p* = 0.006) and T-Cho (*p* = 0.005); multiple comparisons revealed that the PAS group exhibited higher serum TP concentration (*p* < 0.05) and lower T-Cho level (*p* < 0.05). A main effect of AMS was found for TG (*p* = 0.044), whereas no main effects or interactions were detected for HDL-C and LDL-C.

### 3.4. Antioxidant Capacity and Immunoglobulins of Piglets

**Figure 1:**
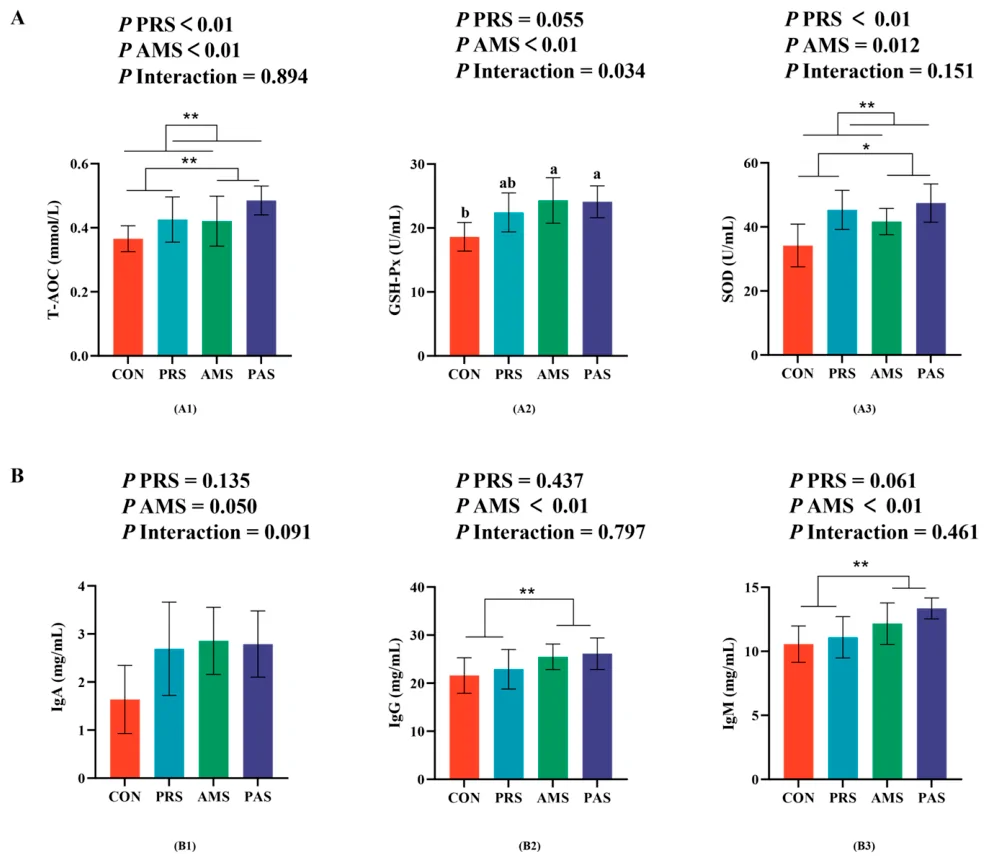
Effects of protease or/and amylase supplementation on antioxidant enzymes (**A**) and immune globulin (**B**). T-AOC, total antioxidant capacity (**A1**); GSH-Px, glutathione peroxidase (**A2**); SOD, superoxide Dismutase (**A3**); IgA, Immunoglobulin A (**B1**); IgM, Immunoglobulin M (**B2**); IgG, Immunoglobulin G (**B3**). Data are presented as mean with SD (n = 3). Significance is shown as * *p* < 0.05, ** *p* < 0.01. Values with different lowercase letters indicate significant differences among groups (*p* < 0.05).

As shown in Figure 1, dietary supplementation with PRS and/or AMS improved serum antioxidant status. The main effects of PRS (*p* < 0.01) and AMS (*p* < 0.01) were observed for T-AOC, and the main effects of PRS (*p* < 0.01) and AMS (*p* = 0.012) were detected for SOD. A PRS × AMS interaction was found for GSH-Px (*p* = 0.034), accompanied by a marginal PRS main effect (*p* = 0.055) and a significant AMS main effect (*p* < 0.01). For immune indices, AMS exerted a main effect on IgA (*p* = 0.050), IgG (*p* < 0.01) and IgM (*p* < 0.01). Multiple comparisons revealed that IgM and IgG concentrations were higher in the AMS groups than in the CON group, indicating enhanced humoral immune status in weaned piglets.

### 3.5. Intestinal Permeability and Fecal Volatile Fatty Acids

**Figure 2:**
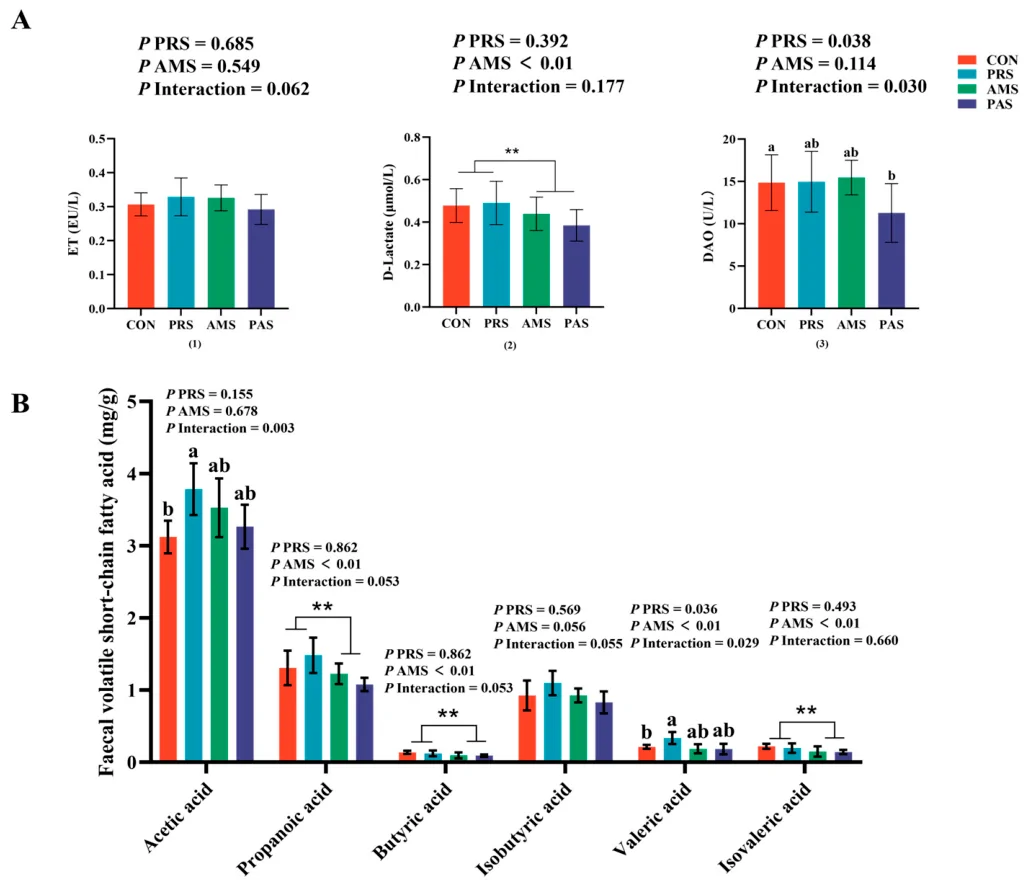
Effects of protease or/and amylase supplementation on intestinal permeability (**A**) and short-chain fatty acids (**B**). ET, endotoxin (**1**); D-Lactate (**2**); DAO, diamine oxidase (**3**). Data are presented as mean with SD (n = 3). Significance is shown as ** *p* < 0.01. Values with different lowercase letters indicate significant differences among groups (*p* < 0.05).

The effects of protease or/and amylase supplementation on intestinal permeability markers and fecal VFAs are shown in Figure 2. D-lactate exhibited a main effect of AMS (*p* < 0.01), while DAO showed a PRS × AMS interaction (*p* = 0.030). The PAS group displayed reduced serum DAO activity (*p* < 0.05), whereas the serum concentrations of ET showed no difference among all groups. For fecal VFAs, propanoic acid, butyric acid, valeric acid and isovaleric acid showed the AMS main effects (*p* < 0.01). Acetic acid had a PRS × AMS interaction (*p* = 0.003), and the PRS group had higher acetic acid and valeric acid concentrations (*p* < 0.05). These results suggested that PRS or/and AMS supplementation improved intestinal barrier integrity and selectively regulated hindgut fermentation.

### 3.6. Fecal Microbiota Analysis

**Figure 3:**
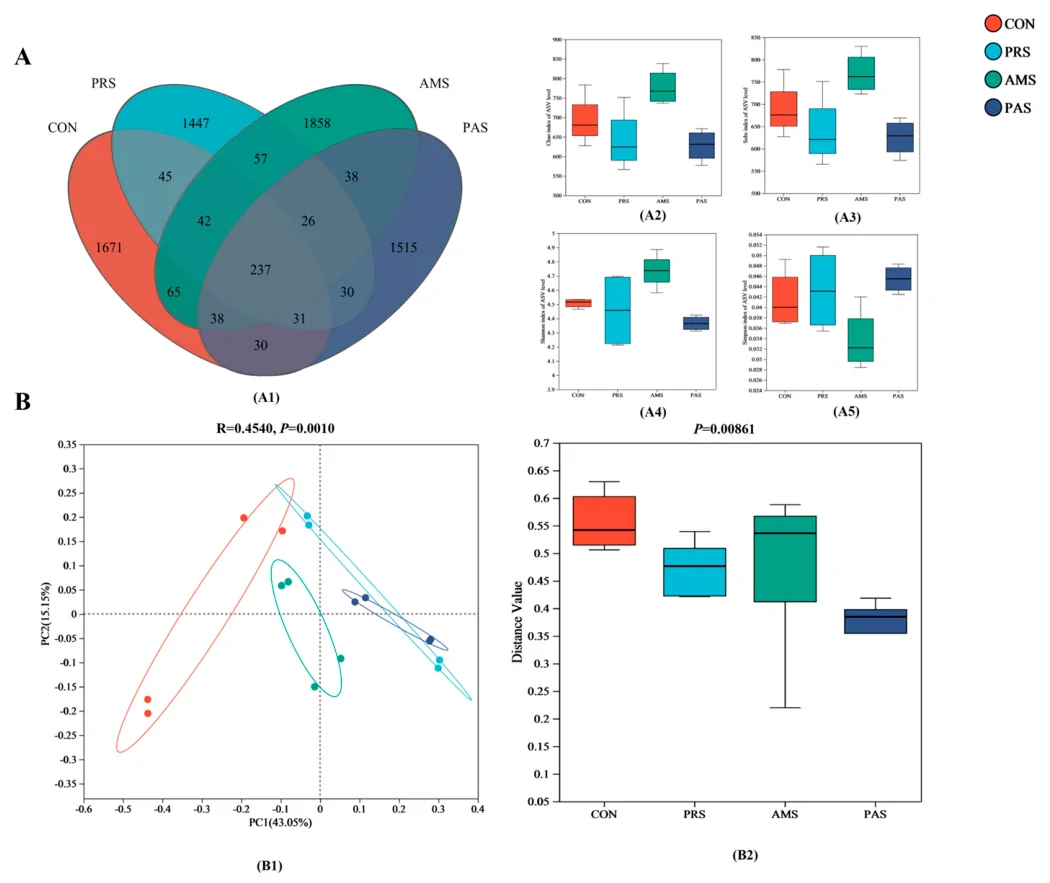
(**A**) Venn diagram of ASV distribution and alpha-diversity indices of gut microbiota among four experimental groups; (**B**) PCoA plot based on Bray−Curtis distance and pairwise boxplot of Bray−Curtis distance for gut microbiota. Venn diagram of ASVs (**A1**). Alpha-diversity analysis based on indices of the Chao (**A2**), Sobs (**A3**), Shannon (**A4**), and Simpson (**A5**) indices. β−diversity analysis based on PCoA (**B1**). β−diversity analysis based on one−way ANOVA (**B2**).

The Venn diagrams revealed that all groups shared 237 amplicon sequence variants (ASVs), and there were 1671, 1447, 1858 and 1515 unique ASVs in the CON group, PRS group, AMS group and PAS group, respectively (Figure 3A). Alpha-diversity, including Chao, Sobs, Shannon and Simpson indices did not differ among groups. However, β−diversity based on principal coordinate analysis (PCoA) revealed distinct clusters among groups. The microbiota structure in all experimental groups deviated from that of the CON group, as further confirmed by an analysis of similarities (one-way ANOVA) (Figure 3B).

**Figure 4:**
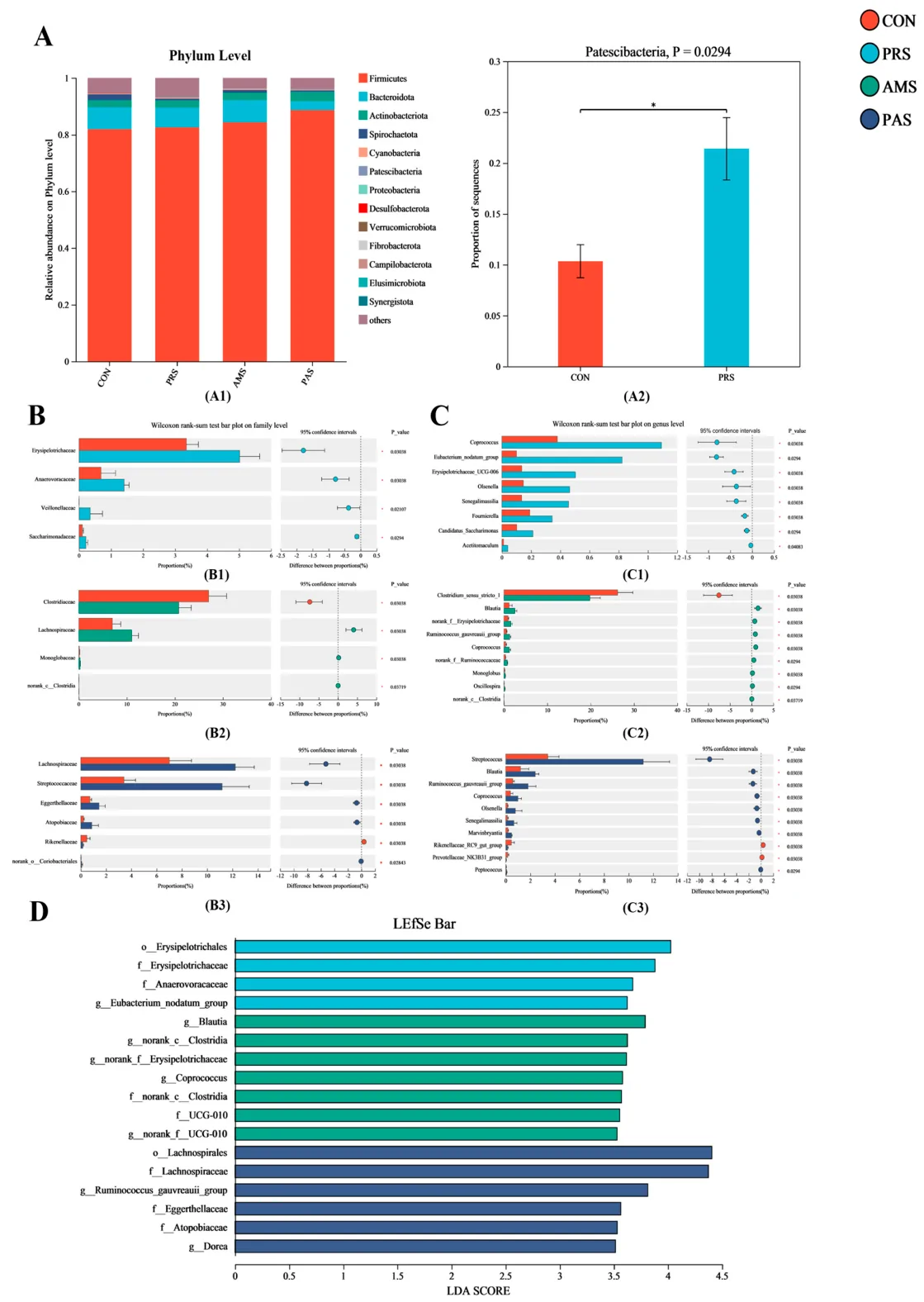
(**A**) Taxonomic composition and differential taxa analysis of gut microbiota across four groups. Phylum-level microbial community composition (**A1**) and the relative abundance comparison of Patescibacteria (**A2**); (**B**) Differential taxa at family level determined by Wilcoxon rank−sum test (**B1**–**B3**); (**C**) Differential taxa at genus level determined by Wilcoxon rank−sum test (**C1**–**C3**); (**D**) LEfSe bar plot showing the discriminative bacterial taxa among groups. CON, control group; PRS, protease group; AMS, amylase group; PAS, protease-amylase combined group. Data are presented as mean with SD (n = 4). Significant differences are displayed in the figures by * *p* < 0.05.

At the phylum level, Firmicutes and Bacteroidetes predominated in all groups, whereas Patescibacteria were enriched in the PRS group (Figure 4A).

At the family level, the PRS group increased the relative abundances of SCFA−producing Erysipelotrichaceae, Anaerovoracaceae, and Veillonellaceae compared with the CON group (*p* < 0.05). The AMS group increased the relative abundances of Erysipelotrichaceae, Monoglobaceae, and Streptococcaceae, while decreasing the relative abundance of the Clostridiaceae (*p* < 0.05); The PAS group increased the relative abundances of the Erysipelotrichaceae and Streptococcaceae, while exhibiting a reduced the relative abundance of the Rikenellaceae, reported as a family linked to malnutrition and dysregulated IgA immunity (*p* < 0.05) (Figure 4B).

At the genus level, compared with the CON group, the PRS group was enriched with *Coprococcus*, *Eubacterium_nodatum*, *Senegalimassilia*, and *Acetitomaculum* (*p* < 0.05), reported with immunomodulatory, fiber-degrading, and acetate-producing functions. The AMS supplementation increased the relative abundances of butyrate- and SCFA-producing taxa, such as *Blautia*, *norank_f__Erysipelotrichaceae*, *Ruminococcus gauvreauii*, Fecalibacterium, *norank_f__R*uminococcaceae, *Monoglobus* and *norank_c__Clostridia* (*p* < 0.05), while reducing the relative abundance of Clostridium (*p* < 0.05). *Streptococcus*, *Blautia*, *Ruminococcus gauvreauii*, *Senegalimassilia* and *Marvinbryantia* are enriched in the PAS group (*p* < 0.05), with decreased relative abundances of *Rikenellaceae_RC9_gut* and *Prevotellaceae_NK3B31* associated with colitis, inflammation, and metabolic impairment (*p* < 0.05) (Figure 4C).

The deviations of microbial communities among groups were screened based on LEfSe analysis (*p* < 0.05 and linear discriminant analysis score, LDA score > 3.5). It was found that Erysipelotrichaceae and Anaerovoracaceae were enriched in the PRS group, *Blautia* and *Coprococcus* were enriched in the AMS group, and Lachnospiraceae and *Ruminococcus gauvreauii* were enriched in the PAS group (Figure 4D).

### 3.7. Metabolomics Analysis

**Figure 5:**
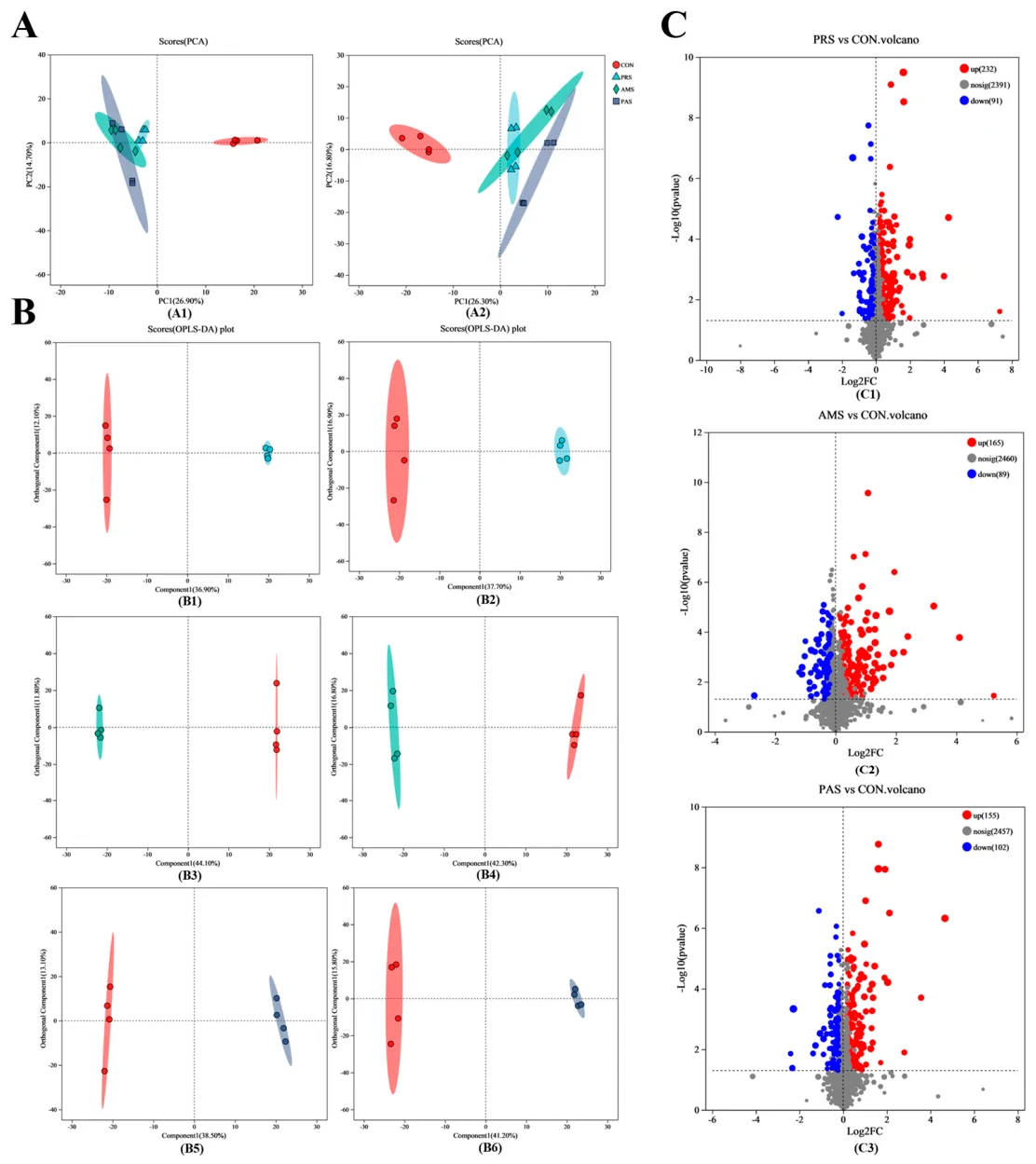
(**A**) PCA score plot, OPLS-DA score plot and volcano plot of fecal metabolites. PCA score plot of fecal metabolites in positive ion mode (**A1**) and in negative ion mode (**A2**). (**B**) OPLS–DA plot of the CON and PRS in positive ion mode (**B1**) and in negative ion mode (**B2**). OPLS–DA plot of the CON and AMS in positive ion mode (**B3**) and in negative ion mode (**B4**). OPLS–DA plot of the CON and PAS in positive ion mode (**B5**) and in negative ion mode (**B6**). (**C**) Differential volcano plot of CON and PRS (**C1**), CON and AMS (**C2**) and CON and PAS (**C3**).

The PCA revealed that PRS, AMS, and PAS supplementation reprogrammed the metabolic profiles of piglets (Figure 5A). Furthermore, OPLS–DA demonstrated distinct, non-overlapping clustering between the CON and experimental groups (Figure 5B), indicating shifts in fecal metabolites. According to Figure 5C, 323 differential metabolites were identified in the PRS group (232 up–regulated and 91 down–regulated, 254 differential metabolites in the AMS group (165 up–regulated and 89 down–regulated) and 257 differential metabolites in the PAS group (155 up–regulated and 102 down–regulated).

**Figure 6:**
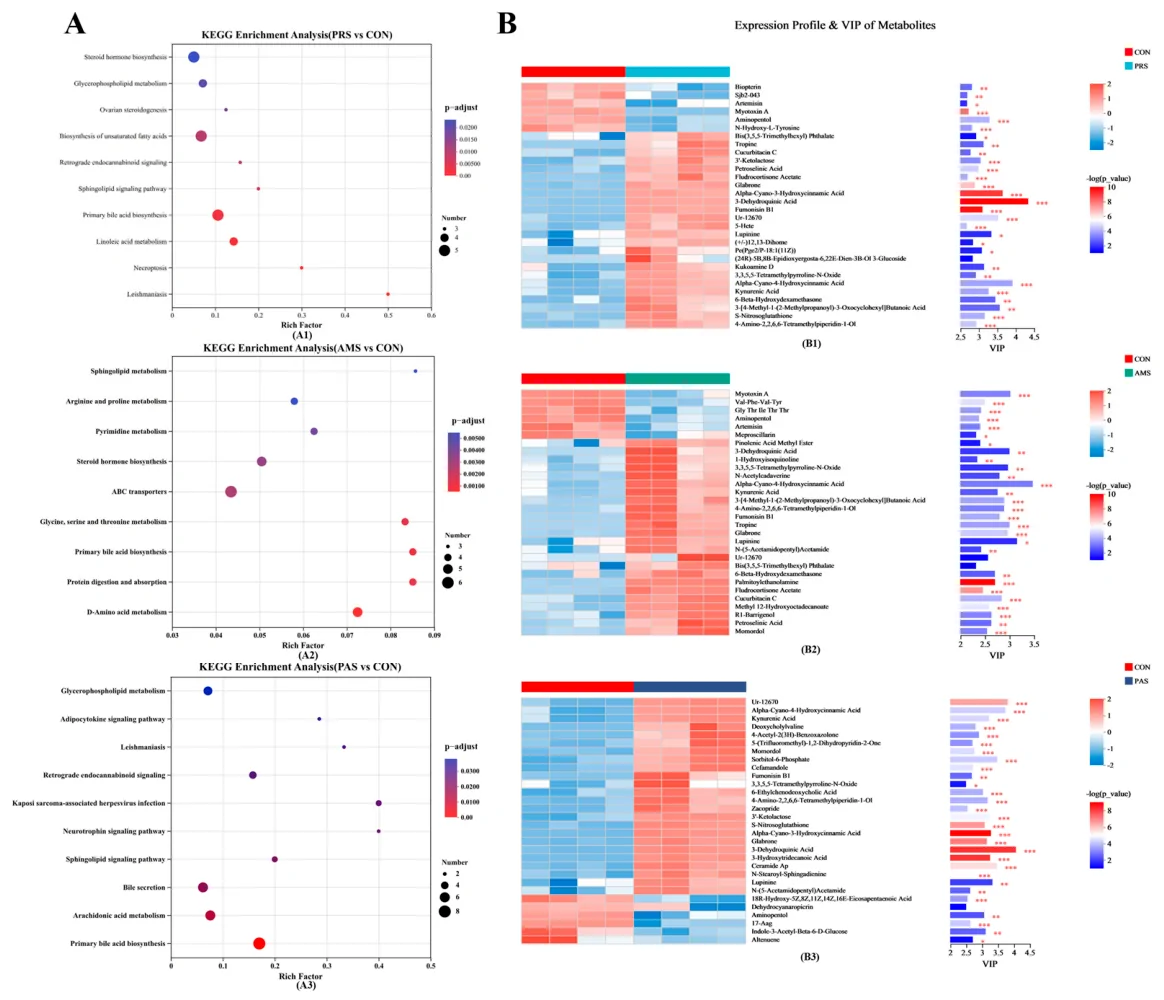
KEGG enrichment bubble diagrams of differential metabolites in the PRS and CON (**A1**), the AMS and CON (**A2**) and the PAS and CON (**A3**). Expression profile and bar charts of VIP of the CON and PRS (**B1**), the CON and AMS (**B2**) and the CON and PAS (**B3**). The size of the bubbles in the figure represents the number of the pathway enriched, and the color of the bubbles indicate the magnitude of enrichment significance based on *p*-values. Red indicates up-regulated metabolites, while blue indicates down-regulated metabolites in the treatment group compared with the CON group. Significance is shown as * *p* < 0.05, ** *p* < 0.01, *** *p* < 0.001

Furthermore, the differential metabolites were imported into the KEGG database to screen the top 30 enriched metabolic pathways. As shown in Figure 6A, the differential metabolites between the PRS and CON groups were enriched in pathways such as primary bile acid biosynthesis, sphingolipid signaling pathway, and biosynthesis of unsaturated fatty acids, while D–amino acid metabolism, protein digestion and absorption, primary bile acid biosynthesis, glycine, serine and threonine metabolism, and arginine and proline metabolism were most enriched in the differences between the AMS group and CON group. Meanwhile, the differential metabolites between the PAS group and CON group were mainly enriched in primary bile acid biosynthesis, arachidonic acid metabolism, bile secretion, sphingolipid signaling pathway, and adipocytokine signaling pathway.

In order to visualize the differences in metabolite patterns more comprehensively, hierarchical clustering analysis was performed based on the top 30 differential metabolites screened between the experimental groups (PRS, AMS, and PAS) and the CON group, using the VIP expression profiles and bar plots. The results are shown in Figure 6B. Compared with the CON group, petroselinic acid, α–cyano–3–hydroxycinnamic acid, kynurenic acid, and S–nitrosoglutathione in the PRS group were up-regulated, while myotoxin A and aminopentol were down-regulated. In the AMS group, kynurenic acid and petroselinic acid were up–regulated, whereas myotoxin A and aminopentol were down–regulated. In the PAS group, kynurenic acid, 6–ethylchenodeoxycholic acid, S–nitrosoglutathione and α–cyano–3–hydroxycinnamic acid were up–regulated, while aminopentol and altenuene were down-regulated.

### 3.8. Correlation Analysis Between Fecal Microbiota and Metabolome

**Figure 7:**
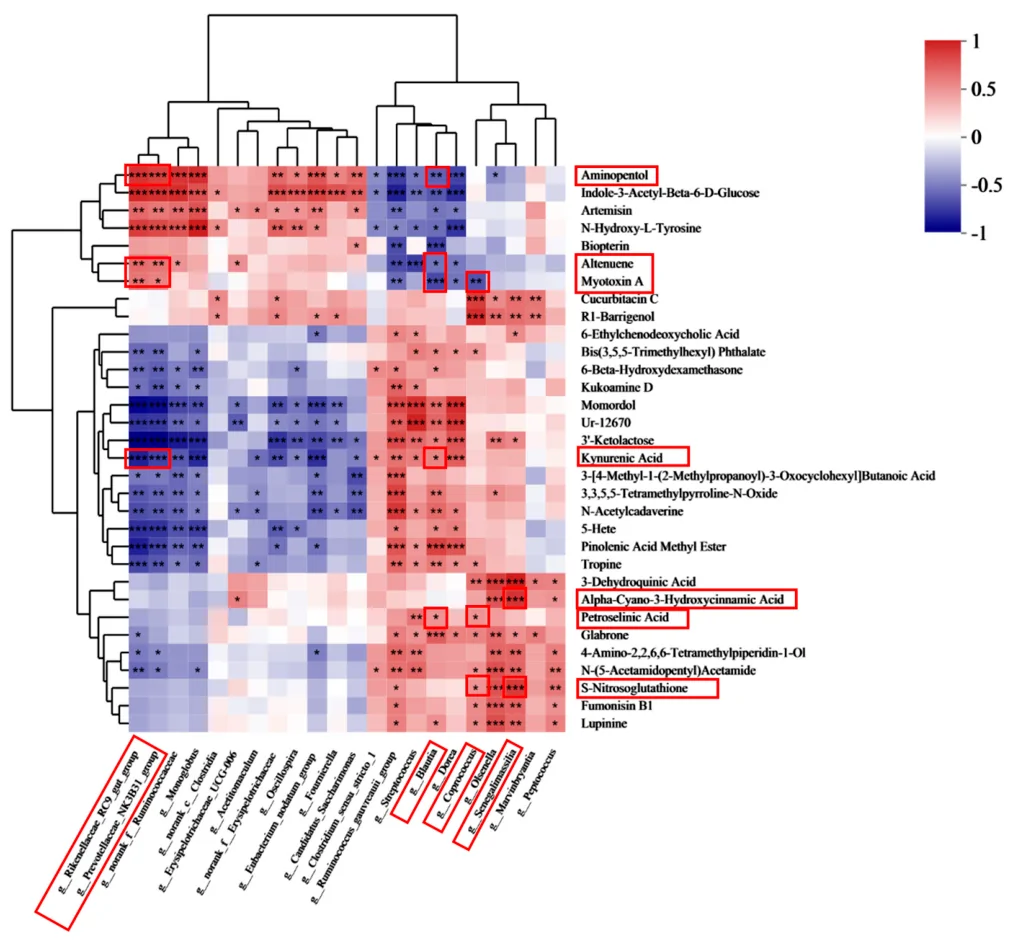
Correlation analysis between fecal bacteria and fecal metabolites. Spearman correlation analysis was performed on all samples pooled across treatments. *p*-values were adjusted for multiple testing based on the FDR method. Significance is shown as * *p* < 0.05, ** *p* < 0.01, *** *p* < 0.001

To further investigate the relationships between fecal microbiota and metabolites, Spearman correlation analysis was performed in all groups (Figure 7). *Coprococcus* and *Senegalimassilia* were positively correlated with α–cyano-3-hydroxycinnamic acid and S-nitrosoglutathione, but negatively correlated with myotoxin A. *Ruminococcus* and *Blautia* were positively correlated with kynurenic acid, petroselinic acid, and S-nitrosoglutathione, while negatively correlated with myotoxin A, aminopentol, and altenuene. *Rikenellaceae_RC9_gut group* and *Prevotellaceae_NK3B31 group* were negatively correlated with kynurenic acid, but positively correlated with myotoxin A, aminopentol, and altenuene.

## 4. Discussion

Weaned piglets exhibit intense basal metabolism and high nutrient requirements. Nevertheless, their underdeveloped digestive systems are highly susceptible to weaning stress, leading to retarded growth or even complete growth arrest [34]. Previous studies have investigated the effects of single supplementation with PRS or AMS, as well as complex enzymes containing both, on animal growth performance, digestive function and other indicators [28,35]. However, the evidence regarding synergistic effects remains limited. These findings suggest that dietary AMS supplementation may compensate for the limited endogenous digestive capacity of newly weaned piglets. The present study showed that AMS supplementation significantly decreased F/G without increasing feed intake, indicating enhanced nutrient utilization.

The apparent digestibility of dietary nutrients serves as an indicator of animals’ ability to utilize nutrients. As shown in Table 3, supplementation with PRS or/and AMS significantly improved the apparent digestibility of EE, as indicated by significant main effects and a significant PRS × AMS interaction. This suggests an additive effect of these enzymes on fat utilization, potentially through improved emulsification or access to lipid substrates. Although PRS directly degrades dietary protein [24,26,28,30], no significant effects of PRS or/and AMS were detected on the digestibility of CP and CF. The lack of effect on digestibility of CP and CF in this study may be due to the adequate endogenous protease activity in piglets at this stage, the specific composition of the basal diet, or the limited accessibility of protease to its substrates, which warrants further investigation.

Serum biochemical indices indicate nutrient metabolism and deposition, serving as key health indicators [36]. UN, a terminal product of protein metabolism, negatively correlates with amino acid deposition efficiency [37], while TP indicates protein anabolism, with higher levels suggesting improved feed conversion and growth [38]. In the present study, dietary supplementation with PRS or AMS significantly reduced serum UN levels, and PAS increased the level of TP in serum, aligning with prior studies [22,28]. Furthermore, reduced T-Cho was observed in the PAS group, indicating that PAS supplementation modulates serum cholesterol metabolism. Collectively, supplementation of PRS or/and AMS can also promote amino acid deposition and body protein synthesis, thereby enhancing growth performance.

Weaning stress typically induces severe oxidative damage and inflammatory activation [2,39]. Key antioxidant indicators, including serum T-AOC, SOD, and GSH-Px, collectively reflect the host’s primary defense against such oxidative challenges [40,41,42]. In our study, PRS or/and AMS supplementation significantly elevated these markers, demonstrating that the enzymes effectively mitigated weaning-induced oxidative stress in piglets.

Immunoglobulins are functional antibodies that bind antigens to reduce pathogen-induced damage, and their serum levels reflect piglets’ immune status. IgG is the predominant immunoglobulin with antibacterial, antiviral and detoxifying capacities [43]. IgA defends against pathogen invasion [44]. IgM is the first antibody responder with bacteriolytic, complement-activating, opsonizing, and agglutinating activities [45]. Exogenous enzymes promote the release of bioactive substances from feed ingredients, including immunomodulatory peptides generated by protein hydrolysis, which activate Toll-like receptor signaling and modulate cytokine secretion [46,47]. This mechanism may support the enhanced humoral immune status observed in weaned piglets receiving enzyme supplementation.

Intestinal barrier protection is particularly critical in weaned piglets because weaning stress can disrupt tight junctions and increase intestinal permeability [1]. D−lactate, a metabolite produced by gut microbiota, and DAO, an intestinal intracellular enzyme, are normally precluded by intact mucosal barrier from serum, serving as markers of intestinal epithelium permeability [48,49,50,51]. The serum level of DAO was significantly reduced by PAS, and serum D−lactate by was significantly reduced by AMS, demonstrating an improvement in intestinal barrier function.

Volatile fatty acids (VFAs), key metabolites of anaerobic fermentation, inhibit pathogenic colonization, enhance tight junction expression, preserve colonic mucosal barrier integrity, and regulate pro-inflammatory cytokine levels [52]. Earlier research found that compound enzyme supplementation with PAS increased colonic acetic acid content and numerically improved propionate and butyrate levels [53]. Consistently, dietary PAS significantly elevated fecal acetic acid concentrations in feces of weaned piglets. Such improvements supply energy and nutrients for intestinal mucosa, reduce intestinal pH, block exogenous pathogen colonization, and sustain stable intestinal barrier function.

The gut microbiota plays a pivotal role in gastrointestinal health of weaned piglets and enzyme supplementation can alleviate dysbiosis. The fecal microbiota analysis revealed that PRS, AMS and PAS altered β−diversity (community composition) without affecting α−diversity (richness), indicating microbial ecosystem reorganization rather than simple diversity changes. At the compositional level, all treatments enriched Lachnospiraceae, *Blautia*, *Coprococcus*, *Senegalimassilia*, and *Marvinbryantia*, which are predominantly butyrate-producing and fiber-fermenting bacteria associated with intestinal barrier function, mucus maintenance, and immune modulation via SCFAs [54,55,56,57,58,59,60,61,62]. Importantly, PAS also reduced potentially detrimental genera, including *Clostridium_sensu_stricto_1*, *Rikenellaceae_RC9_gut*, and *Prevotellaceae_NK3B31*, which are often enriched in dysbiosis and excessive protein fermentation [63,64,65,66]. In summary, dietary PRS or/and AMS can alter the relative abundance of selected fecal bacterial taxa and suppress the propagation of potential harmful bacteria.

The alterations in the intestinal microbiota structure can modulate host metabolism and health. Fecal metabolomics revealed significant enrichment in primary bile acid biosynthesis, sphingolipid signaling, and unsaturated fatty acid metabolism. Up−regulated metabolites in all experimental groups, such as S−nitrosoglutathione, kynurenic acid, and petroselinic acid, are particularly insightful. S−nitrosoglutathione promotes ceramide degradation via nitrosative modification of sphingolipid enzymes and possesses free-radical−scavenging capacity, metabolically explaining the increased serum antioxidant indicators [67,68]. As a metabolite of unsaturated fatty acid metabolism, petroselinic acid activates the AMPK/mTOR pathway to inhibit cell apoptosis and down-regulate IFN−α and IL-6, thereby alleviating intestinal inflammatory damage [69]. Kynurenic acid, a tryptophan metabolite acting through GPR35 receptors, is linked to immune regulation and barrier function, consistent with reduced permeability markers (DAO and D−lactic acid) [70,71,72].

Integrated correlation analysis further supported these associations by demonstrating that health−associated genera, including *Blautia* and *Coprococcus*, were positively correlated with metabolites linked to intestinal health and negatively correlated with potentially harmful metabolites, including myotoxin A, aminopentol, and altenuene, in the PRS, AMS, and PAS groups [73,74]. Although these correlations suggest that enzyme supplementation may influence gut microbiota composition and associated metabolic profiles, they do not establish causality. Therefore, further mechanistic studies are required to elucidate the interactions between dietary enzymes, the gut microbiota, and host metabolism.

## 5. Conclusions

In summary, dietary supplementation with PRS and/or AMS improved nutrient digestibility, antioxidant capacity, immune function, intestinal barrier integrity, and feed efficiency in weaned piglets. These improvements were accompanied by favorable alterations in gut microbiota composition and fecal metabolic profiles, suggesting that modulation of the gut ecosystem may contribute to the observed physiological benefits. Nevertheless, because this study was conducted under controlled experimental conditions, validation under commercial production settings is warranted. Future studies integrating metagenomic, metatranscriptomic, and targeted metabolomic approaches are needed to elucidate the mechanistic interactions within the microbe metabolite host axis underlying these responses.

## Figures and Tables

**Table 1:** Composition and nutrient levels of basal diets.

Item	Content
Ingredients (%)	
Corn	60.25
Soybean meal	15.00
Fermented soybean meal	5.00
Rice bran	5.00
Fish meal	2.50
Whey powder	5.00
Soybean oil	2.50
CaHPO_4_	0.75
Limestone	0.75
NaCl	0.25
Premix ^1^	3.00
Total	100.00
Nutrient levels ^2^ (%)	
DE (MJ/kg)	14.14
CP	17.38
EE	5.64
Ca	0.66
TP	0.61
Lys	1.16
Met	0.37
Met + Cys	0.65
Thr	0.76
Try	0.22

Abbreviations: DE, digestible energy; CP, crude protein; EE, ether extract; TP, total phosphorus. ^1^ The premix provided the following per kilogram of diet: Fe 85 mg; Cu 100 mg; Zn 1200 mg; Mn 3 mg; I 0.35 mg; Se 0.15 mg; VA 10,500 IU; VD3 3000 IU; VE 30 mg; VK3 3 mg; VB1 3 mg; VB2 7.5 mg; VB6 3 mg; VB12 0.03 mg; Nicotinamide 30 mg; D-Calcium pantothenate 15 mg; Folic acid 1 mg; Biotin 0.12 mg; Lys 2 g; Met 0.7 g; Thr 0.7 g; Try 0.2 g; Phytase 0.2 g (500 FTU/kg). ^2^ Nutrient levels are calculated values. Ingredient composition is presented on an as-fed basis.

**Table 2:** Effects of protease or/and amylase supplementation on growth performance.

PRS (mg/kg)	AMS (mg/kg)	Initial BW (kg)	Final BW (kg)	ADG (g/d)	ADFI (g/d)	F/G
0	0	10.38	32.27	521.13	965.28	1.85
	133	10.49	33.75	553.87	966.27	1.75
150	0	10.50	34.03	560.17	991.07	1.77
	133	10.49	34.60	574.06	990.08	1.72
SEM		0.108	0.702	16.447	20.222	0.028
0		10.44	33.01	537.50	965.78	1.80
150		10.50	34.32	567.11	990.58	1.75
SEM		0.076	0.496	11.630	14.299	0.020
	0	10.44	33.15	540.65	978.18	1.81 ^a^
	133	10.49	34.18	563.96	978.18	1.74 ^b^
SEM		0.076	0.496	11.630	14.299	0.020
*p*-value	PRS	0.582	0.099	0.109	0.255	0.096
	AMS	0.665	0.182	0.194	1.000	0.027
	Interaction	0.623	0.538	0.582	0.962	0.320

F/G, feed-to-gain ratio. ^a, b^ Values in a same column with different superscript letters are significantly different (*p* < 0.05).

**Table 3:** Effects of protease or/and amylase supplementation on feed nutrient apparent digestibility.

PRS (mg/kg)	AMS (mg/kg)	CP (%)	EE (%)	CF (%)
0	0	73.94	63.72 ^b^	53.49
	133	77.49	70.94 ^a^	56.77
150	0	79.97	70.456 ^a^	59.95
	133	77.87	70.82 ^a^	54.04
SEM		1.594	1.444	2.198
0		75.71	67.33 ^b^	55.13
150		78.92	70.63 ^a^	57.00
SEM		1.127	1.021	1.554
	0	76.95	67.08 ^b^	56.72
	133	77.68	70.88 ^a^	55.41
SEM		1.127	1.021	1.554
*p*-value	PRS	0.067	0.041	0.412
	AMS	0.658	0.022	0.560
	Interaction	0.101	0.035	0.059

CP, crude protein; EE, ether extract; CF, crude fiber. ^a, b^ Values in a same column with different superscript letters are significantly different (*p* < 0.05).

**Table 4:** Effects of protease or/and amylase supplementation on serum biochemical indexes.

PRS (mg/kg)	AMS (mg/kg)	TP (mg/mL)	TG (mmol/L)	T-Cho (mmol/L)	HDL-C (mmol/L)	LDL-C (mmol/L)	UN (mmol/L)
0	0	39.93 ^b^	0.52	5.68 ^ab^	1.05	1.13	5.40
	133	38.46 ^b^	0.46	5.88 ^a^	1.14	1.20	4.69
150	0	38.61 ^b^	0.51	6.11 ^a^	1.10	1.13	4.63
	133	42.86 ^a^	0.42	5.15 ^b^	1.05	1.14	4.07
SEM		0.988	0.170	0.198	0.038	0.045	0.043
0		39.19	0.49	5.78	1.10	1.16	5.05 ^a^
150		40.74	0.46	5.63	1.07	1.13	4.35 ^b^
SEM		0.698	0.120	0.140	0.038	0.045	0.030
	0	39.27	0.52 ^a^	5.90	1.08	1.13	5.02 ^a^
	133	40.66	0.44 ^b^	5.52	1.10	1.17	4.38 ^b^
SEM		0.698	0.120	0.140	0.054	0.064	0.030
*p*-value	PRS	0.127	0.376	0.452	0.652	0.605	<0.001
	AMS	0.169	0.044	0.061	0.702	0.566	<0.001
	Interaction	0.006	0.684	0.005	0.207	0.655	0.657

TP, total protein; TG, triglyceride; T-Cho, total cholesterol; UN, urea nitrogen. ^a, b^ Values in a same column with different superscript letters are significantly different (*p* < 0.05).

## Data Availability

The data and materials in this study are available from the corresponding authors upon reasonable request.

## Abbreviations

The following abbreviations are used in this manuscript:

ADFI	Average daily feed intake
ADG	Average daily gain
ALB	Albumin
AMS	Amylase
BW	Body Weight
CF	Crude Fiber
CP	Crude protein
d	Day
DAO	Diamine oxidase
EE	Ether extract
ET	Endotoxin
F/G	Feed-to-gain ratio
GLU	Glucose
GSH-Px	Glutathione peroxidase
h	Hour
HDL-C	High-density lipoprotein cholesterol
IFN-γ	Interferon-gamma
IgA	Immunoglobulin A
IgG	Immunoglobulin G
IgM	Immunoglobulin M
IL-1α	Interleukin-1 alpha
LDL-C	Low density lipoprotein cholesterol
PRO	Protease
SCFAs	Short-chain Fatty Acids
SOD	Superoxide Dismutase
T-AOC	Total antioxidant capacity
T-Cho	Total cholesterol
TG	Triglyceride
TP	Total protein
UN	Urea nitrogen
VFAs	Volatile fatty acids
